# Selected Flavonols in Breast and Gynecological Cancer: A Systematic Review

**DOI:** 10.3390/nu15132938

**Published:** 2023-06-28

**Authors:** Dominika Wendlocha, Kamil Krzykawski, Aleksandra Mielczarek-Palacz, Robert Kubina

**Affiliations:** 1 Department of Immunology and Serology, Faculty of Pharmaceutical Sciences in Sosnowiec, Medical University of Silesia in Katowice, 41-200 Sosnowiec, Poland; dwendlocha@sum.edu.pl (D.W.); apalacz@sum.edu.pl (A.M.-P.); 2 Silesia LabMed: Centre for Research and Implementation, Medical University of Silesia in Katowice, 40-752 Katowice, Poland; kamil.krzykawski@sum.edu.pl; 3 Department of Pathology, Faculty of Pharmaceutical Sciences in Sosnowiec, Medical University of Silesia in Katowice, 41-200 Sosnowiec, Poland

**Keywords:** flavonols, gynecological tumors, breast cancer

## Abstract

The consumption of foods that are rich in phenolic compounds has chemopreventive effects on many cancers, including breast cancer, ovarian cancer, and endometrial cancer. A wide spectrum of their health-promoting properties such as antioxidant, anti-inflammatory, and anticancer activities, has been demonstrated. This paper analyzes the mechanisms of the anticancer action of selected common flavonols, including kemferol, myricetin, quercetin, fisetin, galangin, isorhamnetin, and morin, in preclinical studies, with particular emphasis on in vitro studies in gynecological cancers and breast cancer. In the future, these compounds may find applications in the prevention and treatment of gynecological cancers and breast cancer, but this requires further, more advanced research.

## 1. Introduction

Cancer is the leading cause of mortality in both economically developed and underdeveloped countries. Unfortunately, the burden of cancer is expected to increase due to the growth and aging of the population. It has been found that the increased incidence of cancer is the result of a poor lifestyle, including a sedentary lifestyle, smoking, and poor eating habits.

Equally significant for the development of breast and gynecological cancers are reproductive characteristics, including a low number of births and first child births at an older age [1]. Gynecological cancers, including cancers of the cervix, ovary, uterus, vulva, vagina, and fallopian tube, are among the leading causes of cancer-related mortality worldwide. These cancers account for about 10% of all cancers diagnosed in women. Although gynecological cancers are an important public health problem, breast cancer is the most common malignancy in women.

Although significant progress has been made in recent years towards the early detection and treatment of gynecological malignancies, they are still detected at a late stage and have a poor prognosis.

The increased interest in the relationship between the risk of developing cancer of the reproductive organs and dietary factors such as antioxidants has resulted in an increase in scientific research on this topic. However, research results remain limited and inconclusive. A thorough understanding of the links between the consumption of flavonols in the diet and the development of cancer is a reference point for the prevention and inhibition of the development of gynecological cancers and breast cancer [2]. 

In the treatment of breast cancer and gynecological cancers, flavonoids have a high potential of being considered as viable options for new therapeutic solutions and potential candidates for use in chemoprevention. Compounds such as kaempferol (KEM), myricetin (MYR), quercetin (QUE), fisetin (FIS), galangin (GAL), isorhamnetin(IZO), and morin have demonstrated positive results in preclinical studies. These compounds may in the future have applications in both the prevention and therapy for gynecological and breast cancers. The aim of our work was a systematic review of the anticancer activity of selected common flavonols, in preclinical studies, with particular emphasis on in vitro studies in relation to gynecological tumors and breast cancer.

### 1.1. Breast Cancer

**Figure 1:**
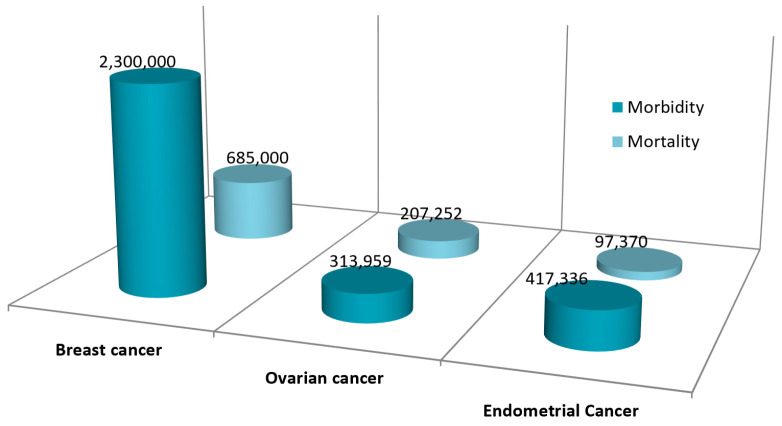
Epidemiology of breast, ovarian, and endometrial cancer worldwide in 2020 [3].

Breast cancer is the most commonly diagnosed cancer in the world, accounting for one in four cancer cases in women. In 2020, as many as 2.3 million new cases were diagnosed, and, unfortunately, the number of patients is still growing. It is estimated that 685,000 women died of breast cancer in the same year (Figure 1), which represents 16% of cancer deaths in women [3,4]. Surgical intervention, supplemented with adjuvant chemotherapy, remains the basic method of treating breast cancer. Current treatment methods focus on targeted therapy and its use for early-stage breast cancer, which depends on ER, PR, and HER2 receptor status; lymph node involvement; and tumor size. In the late stage of the disease, the treatment of choice is systemic therapy based on chemotherapy, hormonetherapy, or immunotherapy [5,6].

### 1.2. Ovarian Cancer

Ovarian cancer is the 5th leading cause of death among women. In 2020, there were approximately 313,959 new cases of ovarian cancer (Figure 1), accounting for 1.2% of all cancer cases, with 207,252 deaths from ovarian cancer [3,7]. The standard treatments for ovarian cancer are chemotherapy and, if possible, surgery to preserve the uterus and ovaries. Complementary treatments include neoadjuvant chemotherapy, adjuvant chemotherapy with cisplatin and paclitaxel, and the use of intraperitoneal chemotherapy [7,8]. The effectiveness of treatment depends on the time of diagnosis, but only 20% of ovarian cancers are diagnosed in stage I or II of the clinical advancement. The chances of successful treatment in stage III and IV of the disease fall below 20% [9].

### 1.3. Endometrial Cancer

Endometrial cancer is a malignant neoplasm of the inner epithelium lining the uterus, which is continuing to increase in incidence and mortality worldwide. In 2020, the incidence was 417,336 worldwide, and the mortality rate was 97,370, making it the sixth most common cancer in women (Figure 1) [3,10]. Endometrial cancer is primarily treated with surgery, while adjuvant treatments include external pelvic radiotherapy, vaginal brachytherapy, chemotherapy, and combined chemotherapy and radiotherapy [11,12].

Studies carried out so far indicate that the patient’s lifestyle, including alcohol consumption, processed food consumption, a lack of physical activity, and obesity, is responsible for the increase in the incidence of these cancers in the population, while only a small percentage of cancers are caused by inherited genetic abnormalities [13].

Epidemiological studies have shown that the consumption of foods rich in phenolic compounds has a chemopreventive effect in the case of cancer. Particular attention should be paid to the wide spectrum of health-promoting properties of flavonoids, including their antioxidant, anti-inflammatory, anticancer, and immunomodulatory effects. The chemopreventive effect of polyphenols on cancer is a consequence of their antioxidant activity, inhibition of the proliferation and survival of cancer cells, inhibition of angiogenesis, and modulation of the immune system, all of which influence the inflammatory process accompanying cancer and the inactivation of pro-carcinogens [14]. In vitro studies have shown that flavonoids can support conventional treatment methods by counteracting treatment resistance in some cancers, e.g., by binding to P-glycoprotein (P-gp) substrate and effecting MRP1 (multidrug resistance protein 1) expression or glucose transporters [15].

## 2. Sources of Flavonoids

Flavonoids are polyphenolic compounds synthesized in plants as bioactive secondary metabolites. The main source of them are fruits and vegetables, as well as cocoa products (cocoa powder and chocolate), black and green tea, and red wine [16].

Vegetables differ in their flavonoid content. Leafy vegetables, cruciferous vegetables, and garlic stand out as having the highest flavonoid content. In particular, green leafy vegetables have demonstrated a higher antioxidant capacity than fruits and root crops. Examples of foods rich in flavonoids include lettuce, celery, potato and onion, which are rich in quercetin, or bok choy, kale, Chinese cabbage, and cauliflower, which are rich in glycosylated quercetin, kaempferol, and isorhamnetin. The flavonoid content of foods is also affected by heat treatment. Research reports that blanched leafy vegetables have an increased concentration of flavonoids compared to fresh vegetables [17].

Some flavonoids play an important role in the development and protection of plants. They are one of the main pigments in plants, imparting a wide range of colors and acting as antioxidants by scavenging reactive oxygen species (ROS) and protecting against damage caused by biotic and abiotic stresses, including UV radiation, the cold, pathogen infection, and insect feeding. They may also act as signaling molecules, attracting insects for pollination [16].

The basic structure of flavonoids consists of two six-carbon benzene rings connected to a three-carbon heterocyclic ring. Based on the degree of the oxidation of the heterocyclic ring and the number of hydroxyl or methyl groups in the benzene ring, flavonoids can be divided into several subgroups (Table 1), including chalcones, flavanones, flavones, flavonols, leucoanthocyanidins, and proanthocyanidins [16,18,19,20].

## 3. Flavonols

**Figure 2:**
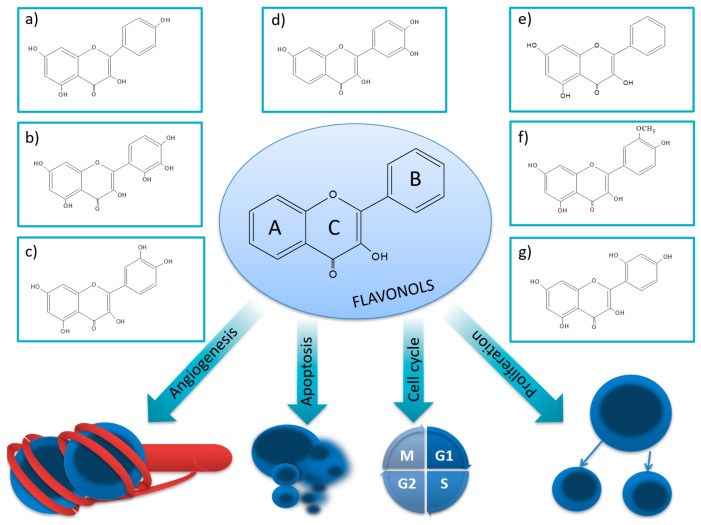
Structure of flavonols and effects on selected mechanisms: (**a**) kaempferol, (**b**) myricetin, (**c**) quercetin, (**d**) fisetin, (**e**) galangin, (**f**) isorhamnetin, and (**g**) morin [17,18,19].

Flavonols (FVLs; IUPAC name: 3-hydroxy-2-phenylchromen-4-one) (Figure 2), which are plant secondary metabolites, is a subgroup of flavonoid organic compounds. Their sources are various fruits and vegetables such as apples, berries, grapes, tomatoes, and onions. Key functions of flavonols include a role in attracting pollinators and protecting plants from UV radiation.

Due to their prevalence in nature, as well as their recognized biological activity, flavonols have attracted the interest of scientists. In recent years, evidence of the chemopreventive effects of flavonols in the fight against cancer has been growing significantly. Detailed attention should be given to the most common in nature: kaempferol, quercetin, myricetin and fisetin, galangin, isorhamnetin, and morin [28].

### 3.1. Kaempferol

Kaempferol (KEM) is one of the most common flavonols in the form of a glycoside. It is a tetrahydroxyflavone, yellow in color with four hydroxyl groups in the 3, 5, 7, and 4′ positions (Figure 2a). KEM occurs in various parts of plants, such as seeds, leaves, fruits, and flowers [29,30]. Its sources include popular foods, including beans, broccoli, cabbage, gooseberries, grapes, kale, strawberries, tomatoes, citrus fruits, apples, and grapefruit.

It is soluble in hot ethanol and alkali ether and slightly soluble in water. It has hydrophobic properties due to its diphenylpropane structure [31,32]. Previous studies on the absorption and metabolism of KEM from food do not provide a clear answer as to how efficient this process is; however, they indicate a strong absorption of the compound from tea and broccoli [33]. Similarly to other flavonols, KEM is absorbed in the small intestine due to its lyophilicity by passive diffusion, facilitated diffusion, and active transport. It is metabolized in the liver by conjugation with glucuronides and sulfates [34].

Among its applications, one can distinguish its anti-inflammatory effects of blocking interleukin 1 beta (IL-1β, interleukin 1β) and tumor necrosis factor (TNF, tumor necrosis factor α) and interfering with the transfer of nuclear factor KB (NF-kB) to the nucleus, thus hindering the synthesis of inflammatory proteins. Studies also report its antidiabetic role by not only acting as a partial competitor of the PPARg agonist receptor (peroxisome proliferator-activated receptor gamma) but also inhibiting the expression of the receptor for advanced glycation end-products (RAGE, receptor for advanced glycation end-products). Its antioxidant activity has also been demonstrated by regulating the expression of heme oxygenase (HO-1, heme oxygenase 1) and mitogen-activated protein kinase (MAPK, mitogen-activated protein kinase) pathways [35,36]. In addition, KEM has cardioprotective, neuroprotective, antioxidant, antibacterial, antifungal, antiprotozoal, and anticancer properties [37,38].

Its anticancer effect has been shown against many types of cancer, including liver, stomach, bladder, and pancreatic cancer and in leukemia [39,40,41,42].

To sum up, the effect of KEM on the development of cancer cells occurs via the modulation of many metabolic pathways in cells. Its effectiveness in inducing apoptosis in cancer cells, inhibiting their proliferation or stopping the cell cycle, has been confirmed [43].

#### 3.1.1. Effect of Kaempferol on Breast Cancer

Breast cancer is an estrogen-dependent cancer. Its development and progression are related to the action of estrogen by a dedicated receptor. Unlike 17B-estradiol (E2), KEMas, a phytoestrogen, inhibits the proliferation of MCF-7 breast cancer cells, eliminating its effects [44]. The relationship between the inhibition of proliferation and the reduction in the expression of the Ki-67 antigen after the treatment of the PMC42 line with KEM has previously been demonstrated [45]. Seung-Heei et al. [44] indicate the different mechanisms of action of KEM in the field of cell proliferation depending on its concentration, emphasizing that a low concentration of the compound may stimulate cell proliferation. In these studies, they point to a decrease in the expression of cyclin D1 and E proteins, and an increase in the expression of p21 protein with a simultaneous decrease in the expression of pIRS-1, pAkt, and pMEK1/2 proteins in MCF-7 cells treated with KEM [44]. Studies by Yi et al. [46] indicate a decrease in the expression of the Bcl-2 protein and an increase in the expression of the Bax protein in the cells of the MCF-7 line, which also leads to the inhibition of tumor proliferation. Similar conclusions were drawn by Lee et al. [47], indicating a downregulation of Bcl-xl expression in KEM-treated VM7LUC4E2 cells. They also showed the stimulation of the apoptosis process by enhancing endoplasmic reticulum (ER) stress after the use of KEM and increasing the expression of p-eiF2alpha and CHOP proteins [47]. Tests conducted with the MDA-MB-468 and MDA-MB-231 lines also showed a strong cytotoxicity of KEM on breast cancer cells [48]. Studies of the local invasion of MDA-MB-231 breast cancer cells indicate a reduced activity of matrix metalloproteinase-3 (MMP-3,matrix metalloproteinase-3) among KEM-treated cells, which in turn significantly reduces the ability of the tumor to spread [49]. A reduction in the activity and expression of MMP-2 and MMP-9 has also been shown [50]. Zhu et al. (Table 2) [51] demonstrated on the lines of BT474 and MDA-MB-231 a decrease in the population of cells in the G1 phase, a simultaneous increase in the population of cells in the G2 phase, and, subsequent, a retention of cells in the G2/M phase [51]. Even at low doses, KEM inhibits the invasion and migration of MDA-MB-231 and MDA-MB-453 cells, blocking the activation of RhoA and Rac1 [52]. Studies conducted on MCF-7 cells on a new derivative, a combination of KEM and 1-deoxynojirimycin, showed a decrease in the expression of cyclooxygenase 2 (COX-2, Cyclooxygenase 2) and the arrest of the cell cycle in the S phase [53].

#### 3.1.2. Effect of Kaempferol on Ovarian Cancer

**Figure 3:**
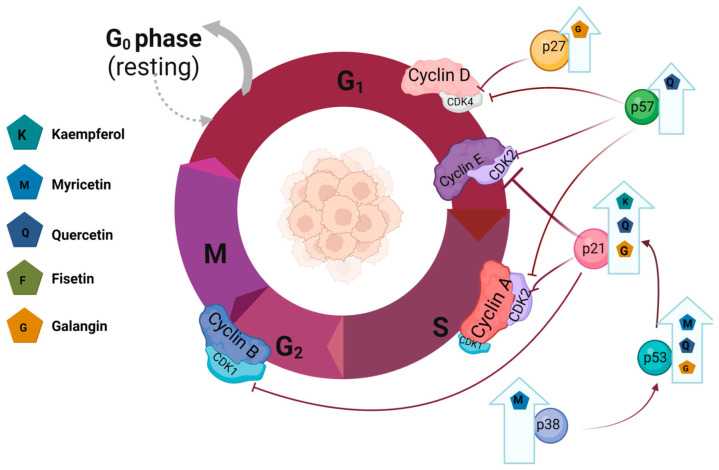
Progression of the cell cycle and its regulation by the cyclins in breast cancer.

KEM is a promising chemopreventive agent. Research so far has indicated its participation in the induction of apoptosis by regulating the expression of pro-apoptotic and anti-apoptotic proteins. Research by Luo et al. [54] indicated an increase in the activity of caspase-3 and -7 in an in vitro model of ovarian cancer comparable to the level after the inclusion of cis-platinum, as well as an increase in the p53 protein, the pro-apoptotic protein Bax, and a decrease in the anti-apoptotic protein Bcl-2. The authors also indicated the involvement of KEM in the induction of the intracellular apoptosis pathway, demonstrating its effect on the regulation of caspase-9 [54]. Similar conclusions were drawn by Yang et al. [55], who also showed an increased expression of caspase-8 in cells of the OVCAR-3 line (Table 3) [55]. The authors showed a significant contribution of KEM to cell cycle arrest in the G2/M phase by increasing the expression of p21 and inactivating the Cdc25C and Cdc2 proteins in the Chk2/Cdc25C/Cdc2 pathway and the Chk2/p21/Cdc2 pathway in A2780/CP70 cells (Figure 3). However, the Chk2 receptor is not directly involved in KEM-induced apoptosis and, instead, mediates an intrinsic pathway with the p53 protein and an extrinsic pathway via the upregulation of DR5 and Fas [56]. In the study by Yinmgmei et al. [57], an increased expression of TRAIL receptors, i.e., DR4 and DR5, was detected on the SKOV3 and OVCAR-3 lines. In addition, an increased expression of ERK1/2, JNK, and p38 was found [57]. As in the case of breast cancer cells, KEM reduced the phosphorylation of pMEK and pERK [55]. Other studies conducted on the OVCAR-3 line indicate synergistic effects of KEM with cisplatin via reduced cell viability by inhibiting the transcription of the ABCC6 and cMyc genes [58]. In addition, the inhibition of proliferation and angiogenesis may occur by reducing the expression of vascular endothelial growth factor (VEGF, vascular endothelial growth factor) (Table 3) [59].

The study by El-kott et al. [60] highlighted the benefits of the simultaneous use of cisplatin with KEM by increasing the levels of the GRP78, PERK, ATF6, IRE-1, LC3II, beclin 1, and caspase-4 proteins, suggesting the possible involvement of autophagy in A2780 cells treated with KEM [60].

#### 3.1.3. Effect of Kaempferol on Endometrial Cancer

Studies conducted on endometrial cancer cells indicate a dose-dependent inhibitory effect of KEM on the cell growth and colony formation of the MFE-280 and HEC-265 lines. Moreover, KEM induces apoptosis accompanied by the upregulation of the Bax gene and the downregulation of Bcl-2 and leads to the blockage of cell division at the G2/M checkpoint [61,62].

KEM’s effect on endometrial cancer is also mediated by the inhibition of the mTOR/PI3K/AKT signaling pathway [61]. These studies are confirmed by Ruan et al., who indicated an increased level of cleaved caspase-9 and caspase-3 in AN3 CA and HEC 1-A cells, the inhibition of the cell cycle in the S phase, and a prolongation of the G2/M phase [63]. KEM treatment inhibited the effect of estradiol on survivin- and estrogen-receptor-induced apoptosis [62].

**Table 2:** The anticancer role of flavonols in breast cancer.

Flavonoid	Cell Line	Mechanism	Result	Refs.
**Kaempferol**	MCF-7	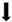 cyklin D1 and cyklin E	Antiproliferative activity	[44]
		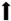 p21	Antiproliferative activity	[44]
		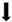 pIRS-1, pAkt, and pMEK1/2,pERK1/2	Antiproliferative activity	[44]
		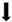 Bcl-2 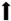 Bax	Induced apoptosis	[44,46]
	VM7LUC4E2	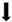 Bcl-xl	Induced apoptosis	[47]
		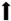 Expression of p-eIF2α and CHOP	Induced apoptosis	[47]
	MDA-MB-231	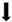 MMP-3	Anti-invasion activity	[49]
		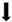 MMP-2 and MMP-9	Anti-invasion activity	[50]
		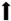 Caspase-3 and caspase-9	Induced apoptosis	[51]
		× RhoA and Rac1	Antimigration activity Anti-invasion activity	[52]
	MDA-MB-453	× RhoA and Rac1	Antimigration activity Anti-invasion activity	[52]
**Myricetin**	T47-D	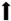 caspase-3	Induced apoptosis	[64]
		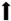 GADD45	Induced apoptosis	[64]
		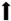 Bax	Induced apoptosis	[64]
	MCF-7	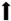 caspase-3, caspase-8, and caspase-9	Induced apoptosis	[65]
		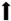 Bax 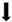 Bcl	Induced apoptosis	[65,66]
		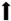 p53	Induced apoptosis	[65]
		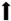 GADD45	Induced apoptosis	[65]
		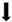 expression MEK1/2	Anti-invasion activity	[66]
		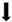 expression p-ERK1/2	Anti-invasion activity	[66]
		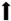 GSK3β	Induced apoptosis	[66]
		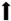 expression β-kateniny	Induced apoptosis	[66]
		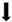 cyklin D1	Induced apoptosis	[66]
	MDA-MB-231	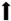 p38	Induced apoptosis	[67]
		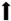 pERK1/2	Induced apoptosis	[67]
	MDA-MB-231Br	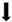 MMP-2 and MMP-9	Anti-invasion activity	[68]
	MDA-MB-468	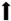 p38	Induced apoptosis	[67]
		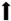 pERK1/2	Induced apoptosis	[67]
	SK-BR-3	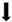 Bcl-2 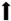 Bax	Induced apoptosis	[69]
		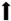 p-JNK i p-38	Induced apoptosis	[69]
		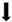 expression p-ERK1/2	Induced apoptosis,	[69]
		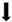 expression p-mTOR/t-mTOR	Induced autophagy	[69]
		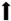 bekliny1 and LC 3-II/I	Induced autophagy	[69]
**Quercetin**	MCF-7	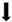 m-TOR and p-m-TOR	Antiproliferative activity	[70]
		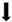 Akt, p-Akt, and p-mTOR/mTOR	Antiproliferative activity	[70,71]
		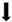 PI3K and p-PI3K	Antiproliferative activity	[70]
		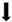 cyklin D1, A, and B	Antiproliferative activity	[70,72,73]
		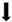 Bcl-2 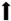 Bax	Induced apoptosis	[70,74,75,76,77]
		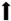 p21, p53, and p57	Antiproliferative activity and Induced apoptosis	[72,73,78,79]
		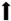 mir-146	Antiproliferative activity	[76]
		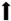 p38MAPK	Inhibited cancer growth	[72]
		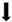 MMP-2 and MMP-9	Anti-invasion activity	[71]
		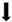 VEGF	Anti-invasion activity	[71]
		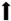 caspase-3, caspase-6, caspase-7 caspase-8, and caspase-9	Induced apoptosis	[73,74,75,76,78,80]
		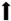 RIPK1 and RIPK3	Induced necroptosis	[74]
		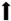 p-JAK2 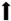 p-STAT1	Promoted apoptosis	[81]
	MDA-MB-231	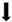 Akt/mTOR.	Antimigration activity and antiproliferative activity	[71,82]
		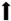 Bax	Induced apoptosis	[76,77]
		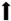 caspase-3	Induced apoptosis	[76,77]
		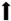 p53 and p21	Antiproliferative activity	[83]
		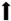 mir-146	Antiproliferative activity	[76]
		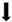 IGF1/IGF1R	Suppressed edepitheliale–mesenchymal transition (EMT)	[84]
		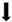 ALDH1A1, CXCR4, EpCAM, and MUC1	Anti-invasion activity	[85]
		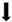 MMP-2 and MMP-9	Anti-invasion activity	[71,84]
		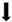 VEGF	Anti-invasion activity	[71]
		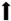 JNK	Induced apoptosis	[83]
	MDA-MB-435	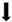 Akt/mTOR	Antimigration activity and antiproliferative activity	[82]
	BT474	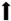 caspase-3 and caspase-8	Induced apoptosis	[86]
	BT 20	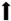 caspase-3, caspase-7, and caspase-8	Induced apoptosis	[80]
**Fisetin**	4T1	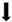 MMP-2 and MMP-9	Anti-invasion activity	[87]
		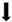 Bcl-2 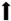 Bax	Antiproliferative activity	[88]
		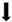 p-PI3K/PI3K,	Antiproliferative activity	[88]
		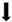 p-Akt/Akt,	Antiproliferative activity	[88]
		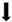 p-mTOR and i-mTOR	Antiproliferative activity	[88]
	MCF-7	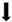 MMP-2 and MMP-9	Anti-invasion activity	[89]
		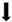 caspase-7, caspase-8, and caspase-9	Induced apoptosis	[90]
	MDA-MB-231	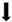 MMP-2 and MMP-9	Anti-invasion activity	[89]
		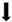 PI3K	Antiproliferative activity	[91]
	MDA-MB-453	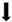 p-Akt	Antiproliferative activity	[92]
		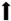 PI3K	Antiproliferative activity	[92]
**Galangin**	T47D	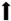 caspase-3 and caspase-9	Induced apoptosis	[93]
		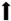 pPERK, pGRP78, pCHOP, and peIF2a	Antiproliferative activity	[93]
		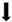 Bcl-2 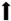 Bax	Induced apoptosis	[93]
	MCF-7	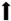 caspase-3, caspase-8, and caspase-9	Induced apoptosis	[93,94]
		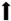 pPERK, pGRP78, pCHOP, and peIF2a	Antiproliferative activity	[93]
		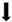 cyclin B1, D3, A, and E	Antiproliferative activity	[94]
		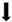 p-PI3K and p Akt	Antiproliferative activity	[94]
		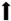 p21, p27, and p53	Antiproliferative activity and induced apoptosis	[94]
		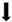 Bcl-2 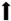 Bax	Induced apoptosis	[93]
		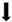 VEGF and ERK1	Anti-invasion activity	[95]
	ER- Hs578T	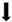 cyclin D3, A, and E	Antiproliferative activity	[96]
	MDA-MB-231	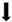 VEGF and ERK1	Anti-invasion activity	[95]
**Isorhamnetin**	MCF-7	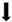 p-AKT, p-mTOR, p-p70S6K, and p-ULK	Antiproliferative activity and induced apoptosis	[97]
		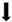 Bcl-2 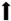 Bax	Induced apoptosis	[98]
		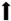 caspase-3	Induced apoptosis	[98]
	MDA-MB-231	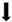 p-AKT, p-mTOR, p-p70S6K, and p-ULK	Antiproliferative activity and induced apoptosis	[97]
	MDA-MB-468	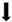 Bcl-2 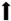 Bax	Induced apoptosis	[98]
		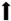 caspase-3	Induced apoptosis	[98]
**Morin**	MDA-MB-231	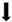 VCAM1	Anti-invasion activity	[99]
		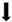 MMP-9	Anti-invasion activity	[100]
		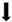 p-AKT	Antiproliferative activity	[100]
		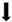 mesenchymal marker N-cadherin	Suppressed epitheliale–mesenchymal transition (EMT)	[100]

x—Blocking the activation.

### 3.2. Myricetin

Myricetin (MYR) belongs to the group of flavonols and is found mainly in plants from the *Myricaceae, Anacardiaceae, Polygonaceae,* and *Pinaceae* families and in some vegetables, fruits, and teas [101]. It is a yellow 3,3′,4′,5,5′,7-hexahydroxyflavone (Figure 2b) occurring in free form and in the form of a glycoside [101,102]. Due to its structural similarity, MYR is also called hydroxy quercetin [103]. MYR is slightly soluble in water and dissolves quickly in an alkaline medium and some organic solvents such as dimethylformamide, dimethylacetamide, and tetrahydrofuran [104]. Due to its low water solubility, MYR is poorly absorbed orally, and at increased doses, absorption by passive diffusion may be observed [103].

The action of MYR is associated with alleviating the symptoms of many diseases such as cancer, heart disease, and diseases with a chronic inflammatory reaction. Thanks to its antioxidant properties and the ability to scavenge free radicals, it has been recognized as a potential modulator of immune reactions and hypertension. It has also been found to be an effective analgesic, anti-allergic, antibacterial, anti-inflammatory, and hypoglycemic agent [101,102,105,106]. A growing number of studies have shown the beneficial effects of MYR on various types of cancer, including liver, skin, pancreatic, and colon cancer. Its operation includes such actions as inhibiting cell proliferation, modifying signaling pathways, inducing apoptosis, and inhibiting angiogenesis and metastasis. It also inhibits key enzymes involved in the initiation and progression of cancer [104].

#### 3.2.1. Effect of Myricetin on Breast Cancer

In vivo studies using breast-cancer-implanted mice showed a reduction in tumor growth among those treated with MYR. In addition, studies showed a significant reduction in the ability to form blood vessels among MYR-treated mice [107].

In vitro studies conducted on the T47-D cell line showed the induction of apoptosis by upregulating the expression of BRCA1, GADD45, caspase-3, and BAX genes after the application of MYR [64]. Nayereh et al. [65], after conducting research on the MCF-7 line, drew similar conclusions, showing an increase in the expression of caspase-8 and -9 and p53 and GADD45 proteins [65]. Additionally, the participation of MYR in many signaling pathways has been demonstrated. MYR inhibits the expression of the PAK1 protein in the PAK1 pathway, reduces the expression of the p-ERK1/2 protein in the ERK1/2 pathway, and activates the GSK3β protein in the GSK3β pathway [66]. Studies conducted on MDA-MB-231 and MDA-MB-468 triple-negative breast cancer cells indicate the participation of MYR in the induction of apoptosis associated with the production of H2O2 and the increase in the phosphorylation of ERK1/2 and p38 proteins [67]. Studies using the MDA-MB-231Br line indicate the inhibition of MMP-2 and MMP-9 protein activity (Table 2) [68]. The arrest of MDA-MB-231 cells in the G2/M phase was also demonstrated after the use of *Bryoniadioica* extract, for which the main component is MYR [108]. Han et al. [69], in a study of an HER-positive breast cancer line, SK-BR-3 showed that MYR has the ability to stimulate apoptosis via the MAPK pathway. Their analysis showed a dose-dependent increase in p-JNK and p-38 levels and a decrease in t-JNK and t-p38 levels with a concomitant decreased expression of p-ERK and increased expression of t-ERK (Figure 3) [69]. An increased amount of vacuoles characteristic for autophagy was observed in MYR-treated SK-BR-3 cells. Further analysis showed an increased expression in beclin1 and LC 3-II/I, with a simultaneous decrease in p-mTOR/t-mTOR expression [69]. Furthermore, Maroufi et al. showed better effects of docetaxel in combination therapy with MYR in inhibiting the growth of the MDA-MB-231 line [109]. Moreover, the studies indicate promising results in the treatment of breast cancer after the use of a preparation made from the synthesis of gold nanoparticles (AuNP) with MYR [110].

#### 3.2.2. Effect of Myricetin on Ovarian Cancer

**Figure 4:**
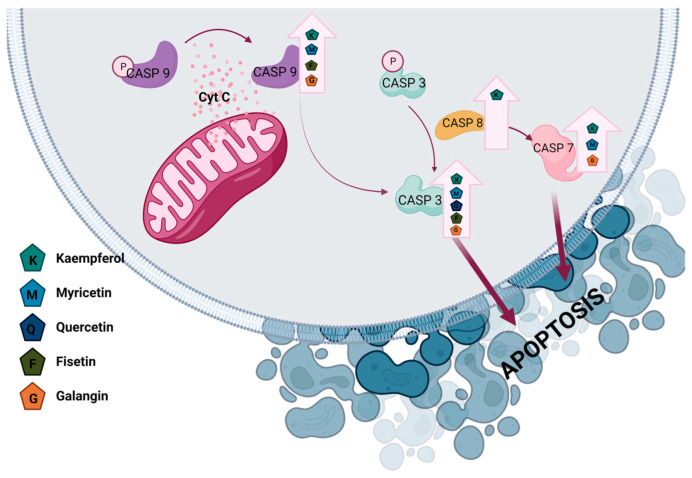
Regulation of caspase in apoptosis signaling pathways in ovarian cancer.

The studies conducted on SKOV-3 cell lines have shown a significant reduction in ROS levels and an increase in p-p38, SAPLA, Bax/Bcl-2, cleaved caspase-3, and caspase-9 with a concomitant decrease in EGFR, MMP2, MMP3, and MMP9 in MYR-treated cells. The stimulation of p38 expression is an important inhibitor of ovarian cancer cell proliferation (Figure 4) [111]. It has been observed that MYR treatment induces apoptosis in A2780 and OVCAR cells, decreases the expression of the anti-apoptotic protein Bcl-2, increases the expression of the pro-apoptotic protein BAX, and induces the extrinsic pathway of apoptosis in OVCAR-3 cells dependent on DR5 [112,113,114]. The studies on cells of the SKOV3 line showed an increase in caspase-3 after treatment with MYR [115]. MYR has also been shown to increase the average fluorescence intensity of GRP-78 in SKOV-3 cells, suggesting that it is involved in ER-stress-related apoptosis [115]. Zhang et al. [114] confirm the significant role of p53 and ERK in the regulation of the apoptosis of ovarian cancer cells. They attribute a special role to the induction of apoptosis in A2780/CP70 cells via the ERK pathway and indicate an increased expression of cleaved caspase-3 and -7 after the use of MYR. In addition, it inhibits the migration of cancer cells [114]. The importance of MYR in the angiogenesis process was also confirmed by inhibiting the secretion of endothelial growth factor (VEGF) and reducing the levels of p-Akt, p-70S6K, and hypoxia-inducible factor 1 (HIF-1α, hypoxia-inducible factor 1), thus indicating the importance of the Akt/ p70S6K/HIF-1α pathway in the process of angiogenesis. MYR has also been shown to increase the levels of p21 and p53 proteins and decrease the level of the oncogene cmyc protein [112,116]. MYR is an inhibitor of protein kinases such as PI3K-PKB/Akt/mTOR, MEK1, Fyn, and JAK1-STAT3 (Table 3) [117].

The effect of MYR on cell cycle regulation has been demonstrated by increasing the percentage of cells in the G0/G1 phase, and its effect on the mechanisms of multidrug resistance (MDR, multiple drug resistance) has been demonstrated by inhibiting the expression of MDR 1 [113,117].

### 3.3. Quercetin

Quercetin (QUE), known as 3,3,4,5,7-pentahydroxyflavone, is one of the most abundant naturally occurring food polyphenols. It exists as a secondary plant metabolite in the form of QUE glycosides (Figure 2c) [118,119]. Its sources in the diet include apples, potatoes, tomatoes, oranges, lettuce, celery, and eggplant [120].

QUE, similarly to other flavonols, is absorbed in the digestive tract and then metabolized by phase II enzymes present in the epithelial cells of the stomach and intestines. The combined metabolites are further processed in the liver and kidneys. Mechanically, the catechol structure (ring B) is methylated at the 3′ or 4′ hydroxyl sites by catechol-O-methyltransferase to produce isorhamnetin and tamarixetine, respectively, which then accumulate in tissues (Figure 2). In vitro studies have shown that QUE metabolites, derived from enterocytes and the liver, serve as antioxidants, inhibiting the oxidation of LDL cholesterol [121].

The bioavailability of QUE is limited due to its poor solubility in water and other solvents. In order to fully exploit the biological potential of QUE, it is necessary to modify its structure or find an appropriate formulation such as nanoparticles or microemulsions [119].

QUE exhibits a wide range of biological activities, such as antioxidant, antiviral, antidiabetic, antifungal, cardioprotective, and anticancer effects [119,122]. The anticancer properties of QUE include effects on cell signaling and the ability to inhibit the enzymes responsible for the activation of carcinogens [123].

#### 3.3.1. Effect of Quercetin on Breast Cancer

Studies conducted on MCF-7 breast cancer cells indicate the effect of QUE both in terms of a decrease in cell viability and growth rate and the ability to form colonies [70,71,123,124,125,126]. These studies have shown significant decreases in the expression of m-TOR, p-m-TOR, PI3K, p-PI3K, Akt and p-Akt, eRα, cyclin D1, and Bcl-2 after the use of QUE, with an increased expression of the Bax protein [70,72,82]. These results suggest the involvement of the PI3K/Akt/Mto signaling pathway in the antitumor effect of QUE [70]. These results are confirmed by other scientists pointing to a significant increase in the expression of caspase-3 and the activity of caspase-6, -8, and -9 after the use of QUE [73,74,75,76,77,78,80,86,127]. Songo et al. [128] point out the increased degradation of ferritin in lysosomes by increasing TFEB nuclear translocation and the activation of lysosomal gene transcription in cells treated with QUE, which also leads to cell death by ferroptosis [128]. Studies indicate an increased phosphorylation of IFNγ-R, p-JAK2, and p-STAT1 proteins while reducing the level of PD-L1 protein, indicating the possible participation of the JAK/STAT1 signaling pathway in the induction of apoptosis and the inhibition of cell growth and differentiation [81]. Tests performed on the cells of the MCF-7 line indicate the arrest of the cycle in the S phase and arresting the cells of the T47D and MDA-MB-231 lines in the G2/M phase [73,82,85,129,130,131]. Moreover, QUE induced the expression of cell cycle regulatory proteins including p16, p21, and p53 (Figure 3) [72,73,78,79,83]. QUE also affects the regulation of the cell cycle via the JNK-Foxo3a signaling pathway, as evidenced by the increase in Foxo3a and JNK activity in cells treated with it [83]. Subsequent studies focusing on the impact of this compound on breast cancer metastases indicate a decrease in the expression and concentration of cell migration marker proteins, including MMP-2, MMP-9, and vascular endothelial growth factor (VEGF) [71,132]. Studies conducted using the MCF-7 and MDA-MB-231 lines indicate the effectiveness of QUE in blocking cellular glycolysis by limiting glucose uptake and lactic acid production and lowering the concentration of proteins related by glycolysis, such as M2 pyruvate kinase (PKM2), glucose transporter 1(GLUT1), and lactate dehydrogenase A (LDHA) [71]. Subsequent studies conducted on the MDA-MB-231 cell line indicate the participation of QUE in blocking the epithelial–mesenchymal transition (EMT—epithelial–mesenchymal transition) of tumors by inhibiting IGF1/IGF1R signaling, QUE inhibits the activation of IGF1R and its other kinases, Akt and Erk1/2, in a dose-dependent manner (Table 2) [84].

QUE also leads to the downregulation of proteins associated with tumorigenesis and progression, such as 1A1 dehydrogenase aldehyde, type 4 C-X-C chemokine receptor, mucin 1, and epithelial cell adhesion molecules [130]. Mostafavi-pour et al. [133] showed a reduction in Nrf2 expression at the mRNA and protein levels [133]. QUE successfully suppressed the expression of anti-apoptotic heat shock proteins (Hsp) including Hsp27, Hsp70, and Hsp 90, high concentrations of which are detected in tumors characterized by high cell metastasis potential and treatment resistance [127].

The studies aimed at elucidating the molecular mechanisms of the cytotoxic effect of the substance indicate an increase in H2AX phosphorylation, which suggests DNA damage caused by QUE [79]. Subsequently, a decrease in EGFR expression, with a simultaneous increase in mir-146 expression, leads to the inhibition of proliferation in MDA-MB-231 and MCF-7 cells [76]. QUE also induces apoptosis by regulating miR-16, 26b, 34a, let-7g, 125a, and miR-605 and reduces the expression of miRNAs such as miR-146a/b, 503, and 194, which are involved in metastases [134]. Khorsandi et al. [74] indicate that not only apoptosis but also necroptosis is involved in the processes of the destruction of cancer cells in which QUE is involved. Researchers showed a significant increase in the expression of necroptosis regulators: RIPK1 and RIPK3 in MCF-7 cells treated with QUE [74].

Combining the action of QUE with already known methods of treatment also brings positive effects [135]. In a study evaluating the apoptosis of MCF-7 cells treated with a chemotherapeutic drug—5-fluorouracil—and QUE, both an improvement in the effectiveness of treatment and an increase in the expression of BAX, p53, and caspase-9 were demonstrated, while the expression of Bcl-2 was reduced [136]. Similar results were obtained with QUE together with docetaxel [137]. In addition, the action of QUE leads to a decrease in the expression of Lef1, responsible for cell resistance to docetaxel, which not only leads to a decrease in the expression of ABCG2, Vim, and Cav1 but also reduces the Smad-dependent activation of the TGF-β (transforming growth factor beta) signaling pathway and, as a result, inhibits the drug resistance of cancer cells [138]. Li et al. [139] indicate a significant reduction in p-glycoprotein (P-pg) mediating the mechanism of multidrug resistance, which significantly improves the response to doxorubicin (DOX) treatment [139]. QUE also acts as an inhibitor of the BCRP protein (breast cancer resistance protein), thus contributing to the abolition of the drug resistance of cancer cells [140]. The combination of QUE in a complex with cerium ions also shows a better effect than QUE alone [141].

The problem with the use of QUE in treatment seems to be the limitations associated with the low solubility in water, bioavailability, permeability, and instability of the compound [142]. The studies on reducing the size of QUE particles to the size of nanoparticles indicate that QUE nanoparticles are more effective in inhibiting MCF-7 proliferation at lower concentrations and do so earlier than QUE [124,143]. Magnetite nanoparticles conjugated with QUE show a high anticancer and radiosynthetic efficacy in MCF-7 cancer cells. This combination enhanced cytotoxicity, cell cycle arrest, immunomodulation, and apoptosis induction, improving the efficacy of the compound [144].

#### 3.3.2. Effect of Quercetin on Ovarian Cancer

The studies conducted on PA-1 cells indicate the effect of QUE in inhibiting the proliferation of cancer cells and their survival by inactivating the PI3k/Akt and Ras/Raf pathways and EGFR expression. QUE also reduces the secretion of the gelatinase enzyme, the proteolytic activity of MMP-2/-9, and the expression of both MMP genes in metastatic PA-1 ovarian cancer cells. The QUE treatment of PA-1 cells also reduces the regulation of linker molecules such as Claudin-4 and Claudin-11 while increasing occludin expression. This is further confirmed by the cell adhesion assay in which QUE reduced the adhesion of PA-1 ovarian cancer cells [145]. The studies conducted by Ren et al. indicate a cell cycle arrest of SKOV-3 ovarian cancer in the G0/G1 phase and a significant decrease in the percentage of cells in the G2/M phase [146]. QUE effectively inhibits tumor growth in cells of this line in a time- and dose-dependent manner [147]. Another anticancer effect also includes the induction of apoptosis in cells treated with QUE by activating both external apoptotic and internal mitochondrial apoptotic pathways [85,123,143,146,148,149].

QUE stimulates the expression of miR-145 in SKOV-3 and A2780 cells in a dose-dependent manner, which may be a factor inhibiting the growth of these cells. This in turn correlates with the increase in the expression of caspase-3 (Figure 4) [148]. QUE enhances ER stress and apoptosis in ovarian cancer cells via the p-STAT3/Bcl-2 axis with an increase in Bax levels and a simultaneous decrease in Bcl-2. An increase in the concentration of caspase-3 and becyclin-1 was also observed, indicating the possible involvement of autophagy under the action of QUE (Figure 4) [150]. The studies conducted by Hasan et al. showed that QUE inhibited the expression of genes encoding key antioxidant enzymes (SOD2, CAT, GPX1, and HO-1), Nrf2 transcription factor, and PI3K/Akt/mTOR signaling pathway kinases (Table 3) [151].

The main difficulty in using QUE as a potential therapeutic agent is its bioavailability. Studies indicate the improvement of the effects of QUE, its stability, and an increased activity by creating nanoparticles in combination with ZnO or the use of graphene oxide together with polyvinylpyrrolidone as carriers of the substance [145,149].

QUE treatment combined with X-ray radiation increased DNA damage in cells, led to cell apoptosis, and increased the expression of Bax, p53, and p21 with a simultaneous decrease in Bcl-2, leading to tumor growth inhibition [152]. Studies on the resistance of many types of ovarian cancer indicate that QUE increases the sensitization of cells by the apoptosis-inducing ligand associated with tumor necrosis factor (TRAIL), thereby leading to the inhibition of tumor growth and an increase in caspase-3 [153]. The simultaneous use of QUE and telomerase inhibitor MST-312 improves the quality of treatment by increasing DNA damage and intensifying apoptosis induction [154].

##### 3.3.3. Effect of Quercetin on Endometrial Cancer

Population studies indicate a lower probability of endometrial cancer among women whose diet is rich in QUE [155]. Studies conducted on RL95-2 endometrial cancer cells showed a reduction in H2O2 cytotoxicity in cells cultured in a QUE medium [156]. Li et al. [157] showed that QUE inhibits the proliferation and migration of HEC-1-A cells, induces cell apoptosis, and affects the cell cycle by arresting cells in the G1 phase. In addition, the antitumor effect of QUE was associated with the induction of ferroptosis in cells with an increase in glutathione peroxidase 4 (GPX4) and a decrease in ferritin light chain expression as well as anti-aconitase 1 [157].

**Table 3:** The anticancer role of flavonols in ovarian cancer.

Flavonoid	Cell line	Mechanism	Result	Ref.
**Kaempferol**	OVCAR-3	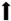 Caspase-3, caspase-7, caspase-8, and caspase-9	Induced apoptosis	[54,55]
		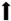 p53 and p38	Induced apoptosis	[54]
		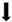 Bcl-2 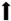 Bax	Induced apoptosis	[54,55,57]
		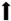 DR4 and DR5	Induced apoptosis	[57]
		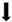 VEGF	Anti-invasion activity	[59]
		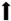 JNK	Induced apoptosis	[57]
		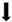 p-MEK and p-ERK	Induced apoptosis,	[55]
	A2780/CP70	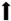 Caspase-3 and caspase-9	Induced apoptosis	[58]
		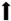 p53 and p21	Induced apoptosis	[58]
		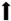 DR5 and FAS	Induced apoptosis,	[56]
		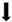 Cdc25c and Cdc2	Cell arrest	[56]
		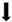 VEGF	Anti-invasion activity and antiangiogenesis	[59]
		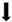 Bcl-2 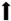 Bax	Induced apoptosis	[58]
	SKOV3	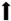 DR4 and DR5	Induced apoptosis	[57]
		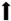 p38	Induced apoptosis	[57]
		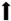 JNK	Induced apoptosis	[55]
	A2780	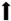 LC3 and beclin-1	Induced autophagy	[60]
		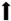 GRP78, ATF6α IRE1α, and PERK	Induced enoplasmic reticulum stress (ER stress)	[60]
**Myricetin**	OVCAR-3	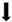 Bcl-2 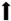 Bax	Induced apoptosis	[112,113]
		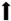 p53 and p21	Induced apoptosis	[112]
		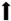 DR5	Induced apoptosis	[112]
		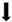 c-myc	Cell cycle arrest	[112]
		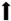 Caspase-3	Induced apoptosis	[113]
		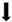 VEGF	Anti-invasion activity, antiangiogenesis	[112,116]
		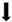 HIF-1α	Antiangiogenesis	[112,116]
		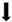 p-p70S6K	Antiangiogenesis	[112,116]
	SKOV3	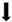 MMP2, MMP3, and MMP9	Anti-invasion activity	[111]
		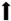 Caspase-3 and caspase-9	Induced apoptosis	[111,115]
		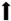 p-p38	Antiproliferative activity	[111]
		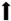 GRP-78	Induced Endoplasmic reticulum stress	[115]
	A2780/CP70	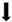 Bcl-2 * 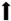 Bax *	Induced apoptosis	[112,114,116]
		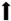 p53 and p21	Induced apoptosis	[112]
		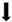 c-myc	Cell cycle arrest	[112]
		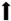 Caspase-3 *, caspase-7 *, caspase-9 *	Induced apoptosis	[112,114]
		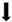 pAkt	Antiproliferation activity	[112,114]
		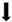 pERK *	Induced apoptosis	[114]
		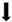 Cycline D *	Cell cycle arrest	[114]
		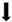 VEGF	Anti-invasion activity, antiangiogenesis	[116]
		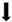 HIF-1α	Antiangiogenesis	[116]
		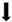 p-p70S6K	Antiangiogenesis	[116]
**Quercetin**	PA-1	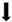 p-PI3k and p-Akt	Antiproliferation activity	[145]
		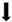 mTOR	Antiproliferation activity	[145]
		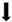 N-Ras and Raf-1	Antiproliferation activity	[145]
		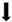 EGFR	Antiproliferation activity	[145]
		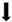 MMP-2 and MMP-9	Anti-invasion activity	[145]
		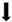 claudin-11 and claudin-4	Antiadherent activity	[145]
	CaOV3	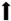 beclin-1	Induced autophagy	[150]
		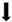 Bcl-2 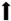 Bax	Induced apoptosis	[150]
		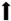 Caspase-3	Induced apoptosis	[150]
	A2780	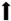 miR-145	Antiproliferation activity	[148]
		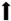 Caspase-3	Induced apoptosis	[148]
	SKOV-3	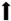 miR-145	Antiproliferation activity	[148]
		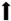 Caspase-3	Induced apoptosis	[148]
		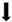 Genes and antioxidant enzymes—Cu, Zn-, Mn-SOD, glutathione peroxidase 1, heme oxygenase 1, and catalase (SOD1, SOD2, CPX1, HO1, and CAT)	Antiproliferation activity	[151]
		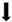 Nrf2 (NFE2L2)	Antiproliferation activity	[151]
**Fisetin**	SKOV-3	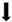 Bcl-2 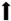 Bax	Induced apoptosis	[158]
		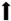 Caspase-3 and caspase-9	Induced apoptosis	[158]
	OVCAR-3	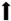 ZBP1, RIP3, and MLKL **	Induced necrosis	[159]
	A2780	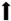 ZBP1, RIP3, and MLKL **	Induced necrosis	[159]
		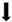 Bcl-2 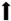 Bax	Induced apoptosis	[160]
		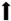 Caspase-3 and caspase-9	Induced apoptosis	[160]
**Galangin**	OVCAR-3	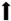 Caspase-3, caspase-7, caspase-9	Induced apoptosis	[161]
		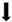 Bcl-2 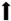 Bax	Induced apoptosis	[161]
		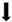 p-Akt, p-p70S6K, and cmyc	Antiproliferation activity	[161]
		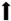 p53 and p21	Induced apoptosis	[161]
		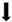 VEGF	Anti-invasion activity and antiangiogenesis	[161]
	A2780/CP70	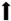 Caspase-3, caspase-7, and caspase-9	Induced apoptosis	[161]
		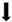 Bcl-2 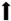 Bax	Induced apoptosis	[161]
		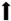 p53 and p21	Induced apoptosis	[161]
		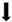 p-Akt, p-p70S6K, and cmyc	Antiproliferation activity	[161]
**Isorhamnetin**	SKOV3	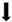 Ki67	Anti-invasion activity	[162]
		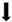 MMP 2 and MMP9	Anti-invasion activity	[162]
**Morin**	A2780	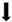 AHNAK, JAG1, and TGFB1	Antimigration	[163]
		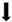 VCAN	Antiadhesion	[163]
		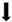 ECM	Antimigration	[163]
	SKOV-3	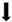 AHNAK, FN1, and STAT3	Antimigration	[163]
		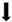 VCAN	Antiadhesion	[163]
		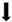 ECM	Antimigration	[163]

* Research conducted with Chinese bayberry leaves; ** research conducted with the z-VAD inhibitor.

### 3.4. Fisetin

Fisetin (FIS) is a bioactive flavonol and has a diphenylpropane structure that contains two aromatic rings joined by an oxidized heterocyclic ring with three carbon atoms, complete with four hydroxyl substitutions and one oxo group (Figure 2d) [164]. FIS is a phytonutrient with low solubility in water, poor absorption from the intestines, and, therefore, low bioavailability. Many researchers indicate that the solubility and bioavailability of FIS can be improved by co-crystallization with caffeine, isonicotinamide, and nicotinamide; complexation with cyclodextrins; and encapsulation into nanoparticles [164]. The main sources of this compound include vegetables and fruits such as strawberries, apples, onions, and cucumbers [165].

Studies indicate that FIS may possess numerous beneficial biological effects, including antioxidant, anti-inflammatory, anti-angiogenic, hypolipidemic, neuroprotective, and anticancer effects. The anticancer activity of FIS is based on the modulation of many different cell signaling pathways including angiogenesis, apoptosis, inflammatory responses, and cell cycle regulation [166,167,168,169].

#### 3.4.1. Effect of Fisetin on Breast Cancer

Studies on the motility and metastasis capacity of cancer cells indicate a dose-dependent reduction in the enzymatic activity and mRNA expression of MMP-2 and MMP-9 cells treated with FIS, as well as its effect on HO-1 induction, which is characterized by anti-apoptotic and antioxidant effects. The authors suggest that FIS may reduce MMP-2 and MMP-9 by acting via HO-1 [87]. The use of combination therapy with FIS with QUE inhibits the proliferation, migration, and colony formation of tumor cells in five types of cell lines, including MCF7, MDA-MB-231, BT549, T47D, and 4T1, and, moreover, in cells of the MCF7 and MDA-MB-231 lines, it reduces the expression MMP-2 and MMP-9 genes [89]. Extended studies on cells of the 4T1 line showed a decrease in the expression of p-PI3K and p-PI3K/PI3K, p-Akt, p-Akt/Akt, p-P70, and p-P70/P-70 and a p-mTOR increase in Bax expression accompanied by a decrease in Bcl- xL [88]. These results are confirmed by studies conducted on the MDA-MB-453 line, which showed a decrease in PI3K activity and Akt phosphorylation at a sufficiently high concentration of FIS [92]. These studies indicate the effect of FIS on tyrosine phosphorylation and HER2 status. The use of the compound led to an inactivation of the receptor and disrupted HER2 signaling by interfering with the PI3K/Akt pathway [92]. Li et al. [91] also confirm the positive effect of FIS in inhibiting the proliferation and metastasis of triple-negative breast cancer in FIS-treated cells. A decrease in the expression of p-Akt and p-GSK-3β was detected with a simultaneous increase in PTEN, which indicates the participation of the PTEN/Akt/GSK-3β signaling pathway in the above-mentioned processes [91]. Subsequent studies conducted on the MCF-7 line with caspase-3 deficiency indicate the participation of FIS in the induction of apoptosis in many ways, including those associated with cell membrane rupture, mitochondrial depolarization, and the activation of caspase-8, -9 and -7 (Table 2) [90]. The effect of FIS on cell cycle inhibition is associated with a significant decrease in the percentage of cells of the MDA-MB-468 line in the G1 phase of the cell cycle with a corresponding increase in the percentage of cells in G2/M. Similar results were obtained in the study of MDA-MB-231 cells, in which there was a significant decrease in the percentage of cells in the G1 phase after the use of FIS [90].

Studies also indicate the inhibition of YB-1 phosphorylation in triple-negative breast cancer cells, thereby inhibiting DNA damage response (DDR) signaling that mediates radiation resistance. Thus, FIS may be a helpful pharmacological agent in highly prognostic breast cancers [170].

Due to the limited solubility of FIS in water and its poor bioavailability, research has been initiated to increase its intracellular concentration by enhancing its penetration into the body. Promising results were obtained after the encapsulation of FIS in polymeric nanoparticles, which increased its stability and storage capacity in the body. FIS nanoparticles show a greater ability to limit tumor growth compared to standard particles. Improving the bioavailability of FIS may increase its usefulness in the treatment of breast cancer [171].

#### 3.4.2. Effect of Fisetin on Ovarian Cancer

Studies conducted on SKOV-3 cells indicated the effect of FIS by increasing tumor cell apoptosis, suppressing proliferation, and inhibiting anti-angiogenic activity [158,172]. Studies conducted with the use of FIS and a caspase inhibitor (z-VAD) indicate a decrease in apoptosis occurring in the FIS environment after the use of the inhibitor. At the same time, it was observed that the inhibition of apoptosis did not significantly increase the growth of the tested cells. Liu et al. [159] suggest that it is not apoptosis that is the basic mechanism of the death of OVCAR-3 and A2780 cells after the application of the compound but necrosis, as evidenced by elevated levels of ZBP1, RIP3, and MLKL proteins, indicating the involvement of the RIP3/MLKL mechanism in the process after the application of FIS together with VAD [159]. The conducted studies indicate an increase in the expression of Bax and Bcl-2 protein genes as well as caspase-3 and -9 after FIS treatment in SKOV-3 cells and in A2780 cells (Figure 4.), which correlated with an increase in apoptosis (Table 3) [160,172].

The simultaneous use of several flavonols, as in the case of the extract of the *Rhusverniciflua* plant containing, among others, FIS and QUE, inhibit AKT-mediated cell proliferation by inducing the apoptosis of paclitaxel-resistant cells and leading to an increase in the level of cleaved caspase-9, -8, and -3 [173].

### 3.5. Galangin

Galangin (GAL), or (3,5,7-trihydroxyflavone), is a naturally occurring flavonoid derived from linden and propolis (Figure 2e) [174]. There are no reports in the available literature on the metabolic profile of GAL. It has been shown that metabolites can significantly affect the efficacy and safety of drugs and may have different biological properties than drugs [175].

GAL exhibits the anticancer activity via several mechanisms having both chemopreventive and therapeutic effects against various types of cancer, such as kidney cancer, hepatocellular carcinoma, or gastric cancer [176,177,178,179].

#### 3.5.1. Effect of Galangin on Breast Cancer

The studies on MCF-7 cells treated with GAL indicate the possibility of inhibiting the activity of glutathione S-transferase P1-1 (GSTP1-1) involved in multidrug resistance [180]. Moreover, GAL inhibits the proliferation and reduces the adhesion of cancer cells and increases the activity of caspase-3 and caspase-9, as well as the Bax protein, which proves its participation in the induction of apoptosis [93,181]. The increase in PERK, GRP78, CHOP, and eIF2a phosphorylation in cells treated with GAL proves its influence on the functioning of the ER stress signaling pathway [93]. These observations are confirmed by other researchers who, in addition to the increase in the activity of caspases, showed a decrease in the level of cyclin D3, cyclin B1, cyclin A, cyclin E CDK1, CDK2, and CDK4 proteins with an increase in the expression of p21, p27, and p53, which proves the influence of GAL on the regulation of the cell cycle (Figure 3) [94,96]. Studies performed on Hs578T cells indicate a blockade of the transition of cells from the G0/G1 phase to the S phase of growth [96]. GAL has the ability to bind in an energetically favorable configuration with the interleukin-6 receptor, leading to interleukin-6 blockade and, thus, influencing the JAK/STAT3 signaling pathway [182].

Malik et al. indicate a decrease in the expression of ERK1 and the VEGF gene in cells of the MDA-MD-231 and MCF-7 lines treated with GAL, thereby inhibiting the angiogenesis process (Table 2) [95].

Considering the low bioavailability and solubility of GAL in water, research is being conducted to find the best possible drug formulation. For this purpose, a new modified cyclodextrin was used, which can be a carrier of GAL and improve the effect of the compound by intensifying apoptosis dependent on caspases [183].

#### 3.5.2. Effect of Galangin on Ovarian Cancer

Studies of A2780/CP70 and OVCAR-3 ovarian carcinoma cells treated with GAL indicate a dose-dependent decrease in cell viability and a significant increase in apoptosis in both lines, which may be associated with increased concentrations of cleaved caspases -3, -9, and -7, increased Bax protein expression, and decreased Bcl-2. Further studies performed on A2780/CP70 and OVCAR-3 cells indicate elevated levels of p53 and p21 proteins and decreased levels of p-Akt, p-p70S6K, and c-myc, suggesting the involvement of GAL in the regulation of tumor cell apoptosis (Figure 4) [161]. The action of GAL also affects angiogenesis induced by cells of the OVCAR-3 line by inhibiting the secretion of vascular endothelial growth factor (VEGF) via the Akt/p70S6K/HIF-1α and p21/HIF-1α/VEGF pathways (Table 3) [116].

#### 3.5.3. Effect of Galangin on Endometrial Cancer

Molecular docking studies confirm the potential effect of GAL on VEGFA and PIK3R1 and indicate its possible use in the treatment of endometrial cancer [184].

### 3.6. Isorhamnetin

Isorhamnetin (IZO) is a plant-derived secondary metabolite of QUE, consisting of two benzene rings and a heterocyclic ring (Figure 2f). Sources include several plant species of Hippophaerhamnoides, Ginkgobilboa, and Opuntia stricte, which are used in traditional medicine, and can be found in several foods, e.g., onions, almonds, and several types of berries [185,186].

For IZO, both transcellular and paracellular transport pathways have been described via various cellular transporters, such as permeability glycoprotein (P-gp), breast cancer resistance protein (BCRP), and multidrug resistance associated protein 2 (MRP2), which are essential for its transport in the intestine [187]. IZO is characterized by a wide spectrum of action, including anti-inflammatory, antioxidant, antibacterial, antiviral, antidiabetic, and anti-obesity effects [185,186].

#### 3.6.1. Effect of Isorhamnetin on Breast Cancer

IZO inhibits the proliferation of MDA-MB-231 breast cancer cells by arresting the cell cycle in the G2/M phase while interrupting the PI3K/AKT/Mtor/P70S6K/ULK signaling pathway. Studies show reduced levels of p-IP3K, p-AKT, p-MTOR, p-p70S6K, and p-ULK [97,98]. In addition, it induces the apoptosis of breast cancer cells in the MCF7 and MDA-MB-468 lines by promoting the activity of the mitochondrial apoptosis signaling pathway and affecting the signaling of the cellular apoptosis signaling pathway, including lowering the expression of Bcl-2 while increasing the expression of Bax and cleaved caspase-3 (Figure 3) [98,188].

Research by Shu et al. [98] indicates an increased expression of cytosolic NF-KB and its inhibitor p65, with a simultaneous decreased expression of nuclear NF-KB, which suggests that the antioxidant effect of IZO may be based on the inhibition of the nuclear translocation of NF-KB (Table 2) [98].

#### 3.6.2. Effect of Isorhamnetin on Ovarian Cancer

IZO regulates the proliferation, migration, and apoptosis of ovarian cancer cell lines in a concentration- and time-dependent manner and may mediate multiple cancer-related signaling pathways [189]. IZO significantly inhibits the expression of MMP2, MMP9 and Ki67, suggesting its involvement in the inhibition of cell adhesion, invasion, and metastasis [162].

Studies on the effect of IZO on the expression of the estrogen receptor (ESR) showed a decrease in the expression of the ESR1 protein with an increasing concentration of the compound in a time-dependent manner, which also affects the reduction in cell proliferation, migration, and invasion [162]. (Table 3).

#### 3.6.3. Effect of Isorhamnetin on Endometrial Cancer

Ye et al. [190] demonstrated that IZO induces apoptosis in Ishikawa cells by inducing the endogenous mitochondrial apoptotic pathway and the exogenous death receptor pathway, promoting the endoplasmic-reticulum-stress-related pathway and activating relevant markers of the UPR response. In addition, IZO affected the expression of MMP2- and MMP9-related proteins in vitro and in vivo and ultimately inhibited the metastasis of cancer cells [190].

### 3.7. Morin

Morin (MOR), or 3,5,7,2′,4′-pentahydroxyflavone, (Figure 2g) is a yellow pigment from various plants, mainly *Moraceae, Rosaceae*, and *Fagaceae*. Its color changes in the open air from yellow to brown. Its main sources include white mulberry, guava, Psidium guava, Osage oranges, apple peels, onions, and almonds. MOR hydrate is soluble in methanol and water, highly soluble in alcohol, slightly soluble in ether and acetic acid, and soluble in aqueous alkaline solutions.

The absorption of MOR from food occurs in the large intestine by initial hydroxylation, which converts MOR-derived compounds into various forms of aglycones. Due to the very short plasma half-life of the derivatives of this compound a better oral bioavailability after intravenous administration is indicated.

MOR is a bioactive compound that exhibits a broad spectrum of biological/pharmacological properties and has a very low cytotoxicity [191].

#### 3.7.1. Effect of Morin on Breast Cancer

The studies on the effect of MOR as a compound inhibiting the adhesion of cancer cells showed the inhibition of VCAM-1 expression and the expression of metalloproteinase-9 (MMP-9) and a reduction in N-cadherin expression on MDA-MB-231 cells, indicating an anti-metastatic effect in breast cancer cells in the participation of the EMT process (epithelial–mesenchymal transition) [99,100]. Additionally, MOR significantly reduced Akt phosphorylation and markedly inhibited the Akt pathway in MDA-MB-231 cells (Table 2) [100].

#### 3.7.2. Effect of Morin on Ovarian Cancer

Studies conducted on cisplatin-sensitive TOV-21G and cisplatin-resistant SK-OV-3 ovarian cancer cells indicate antitumor activity against ovarian cancer cells by reducing cell viability and proliferation as well as increasing apoptosis induction [192].

Research conducted by Nowak et al. showed a significant inhibition of the adhesion and migration potential of A2780 and SKOV-3 cells, accompanied by a decrease in the expression of genes involved in cell migration and motility, including AHNAK, JAG1, TGFB1, ECM molecules, ERBB1 (EGFR), TMEF1, and TMEM132A. In addition, a decrease in the expression of CAMK2N1 and GNG11, which are considered to be EMT regulators, was demonstrated after all the compounds were used. These studies suggest a possible influence of MOR on the regulation of the epithelial–mesenchymal transition process (Table 3) [163].

Cell culture with selected concentrations of MOR and cisplatin shows a synergy of anticancer activity. MOR also affects the sensitization of cells to cisplatin and reduces the expression of galectin-3 both at the mRNA and protein level [192].

## 4. Summary

Based on the studies conducted so far, it has been shown that the consumption of foods rich in phenolic compounds has chemopreventive effects on a number of cancers, including breast cancer, ovarian cancer, and endometrial cancer. Particularly noteworthy is their broad spectrum of health-promoting properties such as antioxidant, anti-inflammatory, anticancer, and immunomodulatory effects.

The chemopreventive effect of polyphenols on cancer is a consequence of their antioxidant capacity, inhibition of proliferation, inhibition of cancer cell survival, inhibition of angiogenesis, and modulation of the immune system, thereby affecting the inflammatory process accompanying cancer and also having an effect on the inactivation of procancerogens.

For this reason, high hopes are currently being pinned on the use of flavonols in anticancer therapy for breast and gynecological cancers, which would allow the creation of new therapeutic solutions aimed at increasing the effectiveness of basic therapy while mitigating the accompanying side effects. Potential “candidates” for use in chemoprevention appear to be compounds such as kemferol, myricetin, quercetin, fisetin, galangin, isorhamnetin, and morin due to their potential therapeutic effects, especially antitumor, anti-inflammatory, antioxidant, and immunomodulatory properties, as demonstrated so far in preclinical studies, particularly in vitro studies. In the future, these compounds may find application in the prevention and treatment of gynecological cancers and breast cancer, but this requires further, more advanced research.

## Figures and Tables

**Table 1:** Classification, structure, bioactivities and sources of flavonoids [21,22,23,24,25,26,27].

Flavonoid	Structure	Bioactivities	Sources	Ref.
**Chalcones**	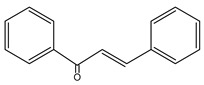	Antioxidant, antimalarial, anti-inflammatory, antimicrobial, antiosteoporosis, antiplasmodial, anticancer, antifungal, and antihyperglycemic	Plants: *Buteamonospermia, Humuluslupulus, Helichrysumrugulosum, Neoraputiamagnifica, Angelicakeiskei, Piperhispidum, Tarennaattenuata,* and *Calythropisaurea*	[21]
**Flavanones**	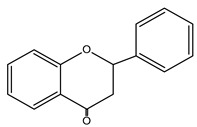	Antioxidant, anti-inflammatory, and anti-ischemic	Citrus (grapefruit, orange, and lemon) and tomatoes	[22]
**Flavones**	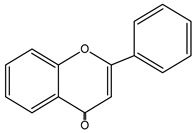	Antitumor, antiviral, antimicrobial, anti-inflammatory, antioxidant, neuroprotective, anti-IR-insulin resistance activity, and hepatoprotective	Plants: *Godmaniaaesculifolia, Tridaxprocumbens, Primulafarinosa* L., and *Chrysanthemummorifolium*	[23]
**Leucoanthocyanidin** ***Flavan-3,4-ol***	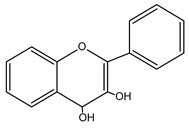	Antioxidation, anti-inflammatory, anticancer, antiviral, and protective cardiovascular properties	Plants: *Acaciapeuce, A. carneorum,* and *A. crombiei*	[24]
**Flavonols**	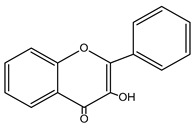	Antioxidant, anticancer, anti-inflammatory, hepatoprotective, neuroprotective, cardioprotective, antimicrobial, and iron-chelating	Berries, citrus fruits, spices, black or green tea, capers, arugula, cabbage, kale, cress, watercress, sea buckthorn, parsley, and carob	[25]
**Proanthocyanidin** ***flavan-3-ol***	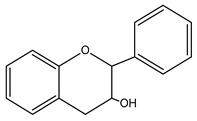	Antifungal, antiviral, anti-inflammatory, anticancer, antiangiogenic, and protective against neurological and heart diseases	Tea, grapes, and wine	[26,27]

## Data Availability

Not applicable.
